# Sleep health, cognitive function, and self‐management skills in middle‐aged adults

**DOI:** 10.1002/alz.087540

**Published:** 2025-01-09

**Authors:** Minjee Kim, Fangyu Yeh, Pauline Zheng, Mary Kwasny, Julia Yoshino‐Benavente, Laura M Curtis, Stacy C Bailey, Morgan Bonham, Britney Sun, Han Q Luu, Patrick Cecil, Prophecy Agyare, Phyllis C Zee, Michael S Wolf

**Affiliations:** ^1^ Northwestern University, Chicago, IL USA; ^2^ Northwestern University Feinberg School of Medicine, Chicago, IL USA

## Abstract

**Background:**

Poor sleep health has been associated with worse cognitive and health outcomes in older adults. Less is known about this relationship in midlife. Thus, we aimed to investigate the relationship between self‐reported sleep health, cognitive function, and performance on common health tasks among middle‐aged adults, as sleep may be a modifiable target to address later life risk of cognitive decline.

**Method:**

English‐speaking adults aged 35‐64 were recruited from an academic general internal medicine practice and federally qualified health centers in the greater Chicagoland area. Multidimensional sleep health (regularity, satisfaction, alertness, timing, efficiency, and duration) was measured by the RU‐SATED questionnaire. Global cognitive function was measured using the Montreal Cognitive Assessment (MoCA); age‐ and education‐adjusted Z‐scores were calculated. Cognitive impairment was defined as MoCA Z‐score lower than one standard deviation below population mean. Performance on common health tasks (e.g. comprehension of print health material, recall of spoken instructions, dosing medications, recall of multimedia education) were assessed using the Comprehensive Health Activities Scale (CHAS). We examined the association between sleep health, cognitive impairment, and health task performance using univariate and multivariable logistic regression. Covariates, selected *a priori*, included age, sex, number of chronic conditions, and depressive symptoms.

**Result:**

A total of 310 participants (mean age 51.2 ± 8.1; 66% female; 39% non‐Hispanic Black, 32% non‐Hispanic White, 21% Hispanic; 54% with 2+ chronic conditions) were included in analyses. The median sleep health score was 8 (interquartile range: 6‐10) and cognitive impairment was found in 11.3%. Poorer sleep health was significantly associated with cognitive impairment after adjusting for *a priori* covariates (adjusted odds ratio, 0.98; 95% CI, 0.96‐0.99; P = 0.007). Both poorer sleep health (ß, 1.11; 95% CI, 0.22‐1.99; P = 0.014) and cognitive impairment (ß, ‐27.7; CI, (‐34.3)‐(‐21.1); P<0.001) were independently associated with poorer performance on health tasks, after adjusting for *a priori* covariates.

**Conclusion:**

Poorer self‐reported sleep health in midlife was associated with a greater likelihood of cognitive impairment, whereas both poorer sleep health and cognitive impairment were associated with lower self‐management abilities to navigate healthcare. Future studies should examine whether sleep‐targeted intervention in midlife can mitigate cognitive later‐life decline and poorer health outcomes.

